# The National Meetings at Old Point Comfort

**Published:** 1894-09

**Authors:** 


### EDITORIAL.

### The National Meetings at Old Point Comfort.

The four National meetings of the dental profession of this country were held at Old Point Comfort the first two weeks of August.

The first was the meeting of the Southern Dental Association.

The second in order was the National Association of Dental Faculties.

The third was that of the Association of State Dental Examining Boards.

The fourth was the annual meeting of the American Dental Association.

In addition to these and during the same time there were various meetings of minor importance. There were alumni meetings, and an Association of the Teachers of Dental Technics was organized, held several sessions, and was put in good working order. There was also held a meeting of the Executive Committee of the World's Columbian Dental Congress, and several other committee meetings upon matters of special professional interest.

The Executive Committee of the Association of Dental Faculties met two days previous to the time of the meeting of the body, for the purpose of preparing the work for the general meeting. A large amount of work was brought before this committee, and of such character and extent as to occupy the time of the committee almost four days in continuous work. The general sessions of the Association occupied two days of very close, earnest work. Much was accomplished by the body that will result in great good to the profession, through its educational institutions. The dental colleges of the country are being constantly improved, and more through the influence of this Association than by any other means. The present status of dental education is due in a large measure to the work of this body. The proceedings of this Association will be published in the October number of the REGISTER, and it is not necessary to anticipate here.

The Southern Dental Association met on Thursday, August 2d, and was largely attended. It has perhaps never had a larger attendance than on this occasion. Quite a number of papers were read and fully discussed; both the papers and the discussions were of great interest. Perhaps as interesting as any part of the proceedings was the consideration of the points presented in the President's address. These were, by a committee appointed for the purpose, arranged and presented to the Association in such a way that each point drew out very interesting discussions. To these points and the discussion had upon them we would ask special consideration by the readers of the REGISTER, they will be found beginning upon page 436 of the present number of the REGISTER.

This Association appointed a committee of three to confer with a like committee from the American Dental Association; the duty of which joint committee shall be to take into consideration the feasibility of uniting the Southern and the American Dental Associations into one organization. The opinion was expressed by quite a number of the members that the time has now come for the unification of these two Associations. This view would seem to be almost if not quite self-evident to every thinking person. The obstacles whatever they may have been in the past, in the way of such union, seem now to be well nigh if not altogether removed. It seems unnecessary that there should be two national bodies of this kind. There is now no more reason for a Southern Dental Association than for a Western Dental Association or an Eastern Dental Association; and, further than this, the combined strength of the two bodies would be much greater and more efficient for good than the divided efforts of the two bodies. Under such a union the mode of procedure and work of the new body would be necessarily somewhat changed from the method of procedure in either body as now presented, and the best interests of the profession would seem to indicate some such change. Both are working upon lines and by methods that have been in operation for many years, and that have become, in a certain sense, worn and threadbare. Change of methods ought, by all means, in the near future to be brought about in each of these bodies whether united or not. By this statement it is not meant that the methods employed have not accomplished desirable results, but the palpable state of affairs well warrants the statement that something in the near future must be adopted different from and in advance of past methods. It is to be hoped that this joint committee will take up this subject in real earnest and give it the consideration its importance demands.

The National Association of Dental Examining Boards met August 4th, and though not as largely represented as it ought to have been, it held sessions of great interest and importance. A subject that received much of its attention was that of uniform laws, of the various States, regulating the practice of dentistry. Certainly this is one of the great needs of our profession, and if this Association should accomplish nothing more in the next five years than to secure uniformity of these laws it would have accomplished a grand result. Another result that has been in some way secured is the more fraternal feeling between this body and the National Association of Faculties; they seem now to be in entire accord, as ought to be the case.

The American Dental Association met on the 7th of August and was largely attended and had interesting and profitable sessions. A goodly number of excellent papers were read and well discussed. New ideas were presented and many old ones put in new dress, which oftentimes is as important as the new.

This body appointed its committee of three to confer with a similar committee from the Southern, looking to the union of the two bodies.

The *bete noir* of the American Dental Association generally turns up when the election of officers comes around. Brethren, can't we have it otherwise in the future? Contention for office will never minister to the welfare of the Association.

Very interesting and instructive clinics were held during these meetings; many valuable things were presented in the way of improvements and new devices, the missing of which was a misfortune to any one who could reach them.

The present number contains a portion of the proceedings of the Southern Dental Association. The October number will contain the remainder, and a portion of the proceedings of the American, and perhaps that of the Association of Faculties.

